# Volatile Organic Compound and Fatty Acid Profile of Milk from Cows and Buffaloes Fed Mycorrhizal or Nonmycorrhizal Ensiled Forage

**DOI:** 10.3390/molecules24081616

**Published:** 2019-04-24

**Authors:** Alessandro Genovese, Andrea Marrazzo, Lucia De Luca, Raffaele Romano, Nadia Manzo, Felicia Masucci, Antonio Di Francia, Raffaele Sacchi

**Affiliations:** Dipartimento di Agraria, Università degli studi di Napoli Federico II via Università 100, 80055 Portici (NA), Italy; draven88@hotmail.it (A.M.); lucia.deluca@unina.it (L.D.L.); rafroman@unina.it (R.R.); nadia.manzo@unina.it (N.M.); masucci@unina.it (F.M.); difranci@unina.it (A.D.F.); sacchi@unina.it (R.S.)

**Keywords:** bovine feed, maize, sorghum, arbuscolar mycorrhizal fungi, volatile compounds, SPME-GC/MS

## Abstract

Seed inoculation of forage crops by arbuscular mycorrhizal fungi (AMF) generally results in higher profitability, but also modifies the chemical composition of silage in terms of increased biomass, protein, and dry matter. Raw milk aroma is affected by the type of feed. This work investigated the influence of ensiled forage obtained by seed inoculation with AMF on the volatile fractions and fatty acid composition of milk. Two experiments were carried out: in the first, buffaloes were fed maize silage, and in the second, cows were fed sorghum silage. The volatile fractions of milk were quantified by headspace solid-phase micro-extraction (HS-SPME), combined with gas chromatography–mass spectrometry (GC-MS), and fatty acids by gas chromatography (GC). The ensiled forage obtained with AMF increased saturated fatty acids (SFAs), and decreased monounsaturated fatty acids (MUFAs) and polyunsaturated fatty acids (PUFAs) in both experiments. The volatile fraction in milk samples obtained from bovines fed mycorrhizal ensiled forage showed an increase of free fatty acids and ketones, responsible for cheesy and fruity odors. Aldehydes, responsible for green and grassy notes, increased only in the milk from buffaloes fed ensiled maize. Our results suggest that inoculation of maize and sorghum seed with AMF, combined with a low rate of fertilizers, leads to ensiled forage that could slightly affect the FA profile and odor quality of milk.

## 1. Introduction

Maize (*Zea mays*) and sorghum (*Sorghum bicolor*) are major worldwide crops and are largely used as feed in livestock farming [1]. A useful method in forage production is the use of arbuscolar mycorrhizal fungi (AMF). These fungi, which form symbiotic relationships with about 80% of herbaceous plant species, including many important crops, have near global distribution [2]. Arbuscolar mycorrhizal fungi decrease plant water stress and increase the acquisition of minerals [2]. Higher levels of phosphorus, nitric oxide, indole-3-acetic acid, sucrose, and glucose in roots are correlated with AMF development [3]. In exchange for these benefits, plants supply AMF with large amounts of carbohydrates [4]. Therefore, AMF can speed up plant growth and reduce fertilizer and water requirements, with economic and ecological advantages. It has been reported that seed inoculation of sorghum and maize forage by AMF, coupled with reduced fertilizer use, generates higher profitability per hectare (+€732 and €670 for sorghum and maize, respectively), and increases net energy (+0.60 and 0.24, respectively) and energy use efficiency (+0.85 and 0.53, respectively) in cattle and buffalo nutrition without affecting milk yield [5]. Although seed inoculation with AMF does not affect the quality of forage, it influences the chemical composition of silage in terms of increased biomass, proteins, and dry matter [5].

Other studies have reported that mycorrhizal plants have different volatile organic compound (VOC) emissions compared to nonmycorrhizal plants, because arbuscular mycorrhizal fungi can strongly influence the metabolism of their host plants [6,7]. Particularly, emissions of the green leaf volatile (*Z*)-3-hexenyl acetate and other plant defensive volatile compounds, like terpenes, were 63% lower in mycorrhizal compared to nonmycorrhizal *Plantago laceolata L*. after an insect herbivore attack [7]. In fact, at the beginning of the process, AMF seem to be able to regulate the methylerythritol phosphate (MEP) pathway of defense-signaling cascades in the plant, and reduce the generation of defense compounds [8]. This occurs because a strong induction of transcript levels of two of the pivotal enzymes of the MEP pathway takes place [9,10]. Another study investigated the effects of AMF on the VOC profile of sorghum (*Sorghum bicolor*) roots. Plants were grown in pots inoculated with either *Glomus mosseae* or *Glomus intraradices* to form mycorrhiza with the roots. The quantity and type of resulting volatiles were dramatically altered by the presence of AMF. Generally, AMF induce plants to emit more alcohols, alkenes, ethers, and acids, but fewer linear-alkanes compared to control plants inoculated with sterile inoculum [11]. There are no studies in the scientific literature about a possible effect of AMF feeds on milk quality and composition.

Milk fat is an important source of nutrients and energy, but it is also crucial, together with VOCs, to the flavor of milk. The fatty acids (FAs) and VOCs of fresh raw milk arise from both animal metabolism and the type of feed. In particular, VOCs are transferred from fodder to milk via the organism (through the lungs or the rumen), or are formed in the organism (in the rumen and/or by metabolic processes in the liver or mammary gland) [12,13]. It has been demonstrated that the first absorption route is the faster one [14,15]. Some studies have reported that the forage type affects the FAs, VOCs, and organoleptic properties of milk [16,17,18,19]. The most volatile free FAs and other VOCs, such as ethyl esters, aldehydes, nitrogen compounds, ketones, alcohols, and dimethylsulphone, represent the main aromatic constituents of milk, and their quantitative differences can explain the different odors that characterize raw milk from different species [20,21,22]. Although the FAs, VOCs, and organoleptic properties in milk have been widely studied in relation to forage type, so far no study has aimed to verify FAs and VOCs in milk when cattle are fed mycorrhizal forage. Since odor plays an important role in the quality of milk and dairy products, it might be very important to know the VOC composition in milk obtained from cattle fed mycorrhizal forage, as it may affect the milk’s sensory quality. This information could be particularly useful for typical dairy products such as Mozzarella di Bufala Campana, a fresh cheese with Protected Designation of Origin (PDO) status that is well-known and exported worldwide.

Therefore, the aim of the present study was to evaluate the combined effect of AMF and low fertilizer application under intensive conditions of crop and livestock farming on the volatile and fatty acid composition of milk in buffaloes (Italian Mediterranean) fed maize silage and cows (Italian Holstein) fed sorghum silage.

## 2. Results

### 2.1. Fatty Acid Composition

#### 2.1.1. Buffalo Milk

Table 1 shows the fatty acid (FA) composition of milk samples obtained from buffaloes fed mycorrhizal (M + m) and nonmycorrhizal (M) maize silage, together with the corresponding level changes expressed as percentages. For each FA, the reported percentage variation was computed by multiplying the ratio between the concentrations detected in M + m and M of milk samples by 100 and then subtracting 100 from the resulting value. A positive percentage value indicates that the FA level was higher in the M + m milk sample, a negative percentage value indicates that the FA level was higher in the M sample. The results show that mycorrhizal silage slightly increased the amount of short and medium chains of saturated fatty acids (SFAs) in the milk, and decreased the monounsaturated fatty acids (MUFAs) and polyunsaturated fatty acids (PUFAs). SFAs (C4:0, C6:0, and C10:0) showed an increase from 5 to 31%, while long-chain saturated fatty acids (C17:0) decreased by about 20%. MUFAs (C17:1, C18:1n9c, C18:1n7, and C24:1) showed a significant reduction from 12 to 26%. The ω-6/ω-3 ratio was higher in the M + m milk samples (+4%), but this difference was not statistically significant.

Finally, PUFAs showed reductions of trans-linoleic acid (C18:2n6tc+ct) and α-linolenic acid (C18:3n3) of about 32% and 24%, respectively. 

#### 2.1.2. Cow Milk

The fatty acid composition of milk obtained from cows fed mycorrhizal (S + m) and nonmycorrhizal (S) sorghum silage is reported in Table 2. Butyric acid (C4:0) and hexanoic acid (C6:0) showed significant increases of 39% and 28%, respectively, in S + m over S milk samples. On the contrary, MUFAs in cow milk showed a significant reduction only for nervonic acid (C14:1) of about 51%. n-3 and n-6 PUFAs did not show significant changes, except for docosahexaenoic acid (DHA) (C22:6n3), which decreased by about 44%. The ω-6/ω-3 ratio was higher in the M + m milk samples (+27%), although this difference was not statistically significant. 

### 2.2. Volatile Organic Compounds

#### 2.2.1. Buffalo Milk

Table 3 shows the headspace concentrations, sensory descriptors, and relative changes of volatile compounds identified in milk samples obtained from buffaloes fed mycorrhizal and nonmycorrhizal maize silage. An increase in ketones, aldehydes, and short and medium free fatty acid concentrations was found in M + m compared to M samples. In particular, the M + m buffalo milk sample showed more 2-heptanone, 2-nonanone, nonanal, decanal, butanoic, octanoic, and decanoic acids in M + m compared to M samples. Finally, acetone and ethyl acetate showed an important decrease in the M + m buffalo milk. From a sensory point of view, 2-heptanone is mainly characterized by cheesy and spicy odors, and 2-nonanone by hot milk, smoked cheese, fruity, and floral odors (Table 3). Based on the concentration data, the odors of 2-heptanone and 2-nonanone increased in M + m milk by about 134% and 46%, respectively. Free fatty acids are mainly responsible for cheesy odors, such as blue cheese, butter, rancid cheese, and goat cheese (Table 3). Butanoic, octanoic, and decanoic acid odors increased in M + m by about 27%, 49%, and 28%, respectively. On the other hand, aldehydes are mainly responsible for grassy, green, and animal notes (Table 3). An increase of these odors was particularly marked in M + m buffalo milk, e.g., by about 88% for nonanal. In contrast, the sticky and sweet odor of ethyl acetate was completely absent in M + m milk. 

#### 2.2.2. Cow Milk

Table 4 shows the headspace concentrations, sensory descriptors, and relative changes of volatile compounds identified in milk samples obtained from cows fed mycorrhizal and nonmycorrhizal maize silage. The cow milk showed a higher increase of most free fatty acids and only one ketone (2-nonanone). 2-Butanone and ethyl acetate compounds showed slight decreases. From a sensory point of view, based on the concentration data, the cheesy odor of free fatty acids increased in S + m cow milk by about double, with a maximum increase of 279% for butanoic acid and a minimum increase 37% for tetradecanoic acid (Table 4). The hot milk, smoked cheese, and fruity odors of 2-nonanone increased by about 88%. By contrast, the varnish odor of 2-butanone decreased by about 17% (Table 4). As reported, for buffalo milk, the sticky and sweet odors of ethyl acetate were completely absent in SM+ cow milk (Table 4). 

## 3. Discussion

Typically, fat in both buffalo and cow milk contains about 70% SFAs and 30% MUFAs and PUFAs [23]. Our buffalo and cow milk samples had 10% more SFAs when compared to the aforementioned study, with lower levels of MUFAs and PUFAs. Different authors have reported changes in the FA composition of milk and dairy products as a function of the feeding system [24,25,26]. Maize silage is rich in linoleic and oleic acids and poor in linolenic acid. It has been reported that, compared to a hay-based diet, a diet rich in maize silage and concentrates decreases the concentration of branched-chain FAs [27]. An intensive feeding system that uses a highly concentrated and stored forage-based diet and increased milking frequency could further reduce the concentration of PUFAs [28,29]. Sometimes, to optimize the balance of FAs in milk fat in intensive feeding systems, bovines are fed fish oils or oilseed lipid supplements, which are rich in unsaturated FAs, in order to increase the PUFA level in their milk [30,31]. Although the content of fat has been found to be similar in mycorrhizal and nonmycorrhizal milk samples for the same feeding tests [5], the differences in FA composition presented here are probably related to the higher number of leaves formed in mycorrhizal plants [5,11], which could influence the fermentation and FA composition of the silage. However, there is a scarcity of studies on feeding tests with mycorrhizal forage and the differences that affect the FA composition in milk, which makes these differences difficult to understand. 

The differences found in FAs could also affect the texture of dairy products. In fact, some dairy products characterized by higher SFAs are reported to be less soft and more viscous than dairy products characterized by higher levels of MUFAs and PUFAs [32]. 

FA composition could also indirectly influence the aroma of dairy products. In fact, they act as precursors of other important aroma components, such as methylketones, alcohols, aldehydes, and esters. Methylketones are the result of the enzymatic oxidation of free fatty acids to β-ketoacids and their consequent decarboxylation, with the loss of one carbon atom, leading to alkan-2-ones [33]. In both buffaloes and cows, mycorrhizal milk samples showed a trend of a higher n ω-6/ω-3 ratio than nonmycorrhizal. This difference was not statistically significant, but for both mycorrhizal and nonmycorrhizal milk samples, the ω-6/ω-3 ratio was above the recommended limit of 5:1. The higher ratio for M + m samples can be explained by higher amounts of linoleic acid and lower amounts of linolenic acid in contrast to M samples. Even if both ω-6 and ω-3 FAs are essential for humans, because they cannot be formed de novo, the correct ratio is need for human health. In fact, a study conducted by Strandvik [34] demonstrated that a greater consumption of ω-6 could be one of the main causes of childhood obesity. 

The higher level of alkan-2-ones, as well as ethyl acetate, found in buffalo milk compared to cow milk is in agreement with the data reported in the literature [20]. It is probably related to the greater quantities of FAs present in buffalo milk. The mycorrhizal forage is responsible for increased VOC emissions from the root [11]. Among VOCs produced by AMF in the root, there are some organic acids that can be transferred from forage directly into milk through the rumen and the respiratory tract [12,13]. The latter route has been reported to be faster [14,15], and could explain the higher level of VOCs found in milk obtained from animals fed mycorrhizal forage.

In conclusion, seed inoculation of maize and sorghum plants with AMF is a good alternative for producing ensiled forage, as it slightly affects the quality of milk in terms of FA and VOC composition. Further sensory studies are needed to confirm our findings and better understand whether and to what extent mycorrhizal forage affects the sensory properties of dairy products. From an application point of view, this information could be of interest to milk producers, in order to protect and monitor the PDO quality of dairy products.

## 4. Materials and Methods

### 4.1. Experimental Design, Forage Species, and Mycorrhizal Treatment

The tests were conducted at two dairy farms (named A and B) in the Campania region of southern Italy, as reported by Uzun et al. [5]. Farm A was a buffalo (Italian Mediterranean) dairy farm (154 ha used as an utilized agricultural area (UAA; 368 buffaloes), located on an irrigated flat area at sea level (40°31′N, 14°57′E, 13 m a.s.l.). The climate is Mediterranean–maritime with relatively dry summers (84 mm) and average annual rainfall of 988 mm, with monthly average temperatures ranging from 23.6 °C (August) to 9.0 °C (January). Farm B was a dairy cattle farm (60 ha used as a UAA, 60 cows), situated in the foothills of the Apennine mountains (41°16′N, 15°05′E, 760 m a.s.l.), under Mediterranean, subcontinental climate conditions. Average annual rainfall (1001 mm) is concentrated in fall and winter and summers are generally warm (15–20 °C). Due to the different environmental and agricultural context of the experimental sites, two forage species were investigated: maize (*Zea mays L*., cv Indaco, class FAO 600, Limagrain, Busseto, PR, Italy) and sorghum (*Sorghum sudanense Piper*, cv Hermes, medium–early hybrid Hermes Sudangrass-Sudangrass, Padana Sementi Elette, Tombolo, PD, Italy) for farms A and B, respectively. Seeds of both forages were inoculated on-farm (May 2014) with the same microbial complex (Aegis, Italpollina, Rivoli Veronese, VR, Italy) containing the mycorrhizal fungi *Glomus intraradices* (700 spores/g) and *Glomus mossae* (700 spores g^–1^). The microbial product, mixed with a green adhesive powder and a solution containing vegetal amino acids and peptides, was sprayed on sorghum and maize seeds in a cement mixer in the quantity of 20 g inoculum kg^−1^ seed.

### 4.2. Maize Test on Farm A

Mycorrhizal and nonmycorrhizal maize seeds were sown on 7 June 2014, on two plots of 1.5 ha each per treatment. The soil had a clay loam texture (clay 64.6%, silt 18.4%, sand 17.0%) and alkaline conditions (pH 8.2, 1:5 soil/water extract), showing the following properties before seeding: total N 2.3 g kg^−1^ (Kjeldhal method), organic matter 36.0 g kg^−1^ (Walkley and Black method), total carbonate 30.0 g kg^−1^ (manometric method), available phosphorus pentoxide (P_2_O_5_) 30.0 mg kg^−1^ (Olsen method). The maize control treatment (M) was nonmycorrhizal maize that received the full dose of P and N (i.e., N 250 kg ha^−1^, P 100 kg ha^−1^) and calculated on the basis of expected uptake (3.9 kg N t^−1^ yield, and 15 P_2_O_5_ t^−1^ yields) and yield (65.0 t ha^−1^) suggested by the Department of Agriculture of Campania Region [47]. The doses adopted were similar to those used by farmers in local practice. The full dose of P_2_O_5_ was applied at seedbed preparation, while one-third of N was applied at the time of sowing and the remaining two-thirds at fourth-leaf stage, or growth stage (GS) 14 according to the BBCH (Biologische Bundesanstalt, Bundessortenamt und CHemische Industrie) scale [48]. The experimental maize test (M + m) was mycorrhizal maize that received only half the dose of N (125 kg ha^−1^). The maize was irrigated when required, for a total irrigation volume of 600 m^3^ ha^−1^. M + m and M maize were harvested on 25 September 2014 at the kernel dent ripening phase (GS85), and put into 4 silo bags: one per plot, two per treatment. The forage was chopped to 1.50 cm theoretical length of cut using the same harvester. Forty lactating buffaloes were divided into groups differing only in the use of inoculated (M + m group) or control (M group) maize silage (the experimental test was 16 days long). The groups were balanced for number (20), parity (2.2 ± 0.6 and 2.3 ± 0.6 for M + m and M groups, respectively), days in milk (98 ± 19 and 98 ± 18 days), and milk production (12.0 ± 2.4 and 12.0 ± 4.9 kg d^−1^ per head). Ingredients of diets (kg per day) fed to lactating buffaloes were as follows: mycorrhizal silage (20 and 0, for M + m and M groups, respectively), nonmycorrhizal silage (0 and 20), meadow hay (4.5 and 4.5), mash maize (3.0 and 3.0), concentrate (a mixture based on soybean, maize, sunflower, and barley meals) (4.5 and 4.5), and a mix of vitamins and minerals (0.3 and 0.3). The chemical composition of the diets expressed as g kg^−1^ of dry matter was as follows: crude protein 141 and 140, ether extract 26.3 and 26.4, neutral detergent fiber 380 and 373, acid detergent fiber 254, and 274, and UFL (Unité Fourragère Lait) 870 and 870. After 10 days of adapting to the diets, according to Filho et al. [49], dry matter intake was measured over six consecutive days on a group basis. Milk samples were automatically collected twice daily, in the morning (05:00 h) and the evening (17:00 h). The milk yield was 11.1 and 11.3 kg d^−1^ per head, respectively, for the M + m and M groups. The animals were housed in 2 barns where they were free to move. Each barn was furnished with a feed manger, automatic drinker, and outdoor paddock. The animals were handled in a similar way in terms of feeding (once daily at 07:00 h).

### 4.3. Sorghum Test on Farm B

Mycorrhizal and nonmycorrhizal sorghum seeds were sown on 3 June 2014 on four plots, two per treatment, 1.5 ha each. The soil was sandy-silty-loam (sand 57.4%, silt 21.3%, clay 21.3%), alkaline (pH 8.3, 1:5 soil/water extract), with the following characteristics at the beginning of the experiment: total N 1.2 g kg^−1^, organic matter 10.0 g kg^−1^, total carbonate 70.7 g kg^−1^, available P_2_O_5_ 3.3 mg kg^−1^. The sorghum control treatment (S) was nonmycorrhizal sorghum that received a recommended dose of P_2_O_5_ and N (i.e., P_2_O_5_ 50 kg ha^−1^, N 120 kg ha^−1^), calculated as described previously (expected uptake 3.0 kg N and 1.25 kg P_2_O_5_ per t of yield; expected biomass 40.0 t ha^−1^). The full dose of P_2_O_5_ was applied at seedbed preparation, while half of the N was applied at the time of sowing and the remaining half at fifth-leaf stage (GS 15). The experimental sorghum test (S + m) was mycorrhizal sorghum that received only half the dose of N (60 kg ha^−1^). Sorghum grew under rainfed conditions, and total rainfall during the crop cycle was 196 mm. Sorghum was harvested at flowering stage (GS 61), on 30 August 2014. The S + m and S forages were ensiled in bales wrapped in polyethylene film. Twenty-eight lactating cows (Italian Friesian cattle) were divided into groups differing only in the use of inoculated (S + m group) or control (S group) sorghum silage (the experimental test was 16 days long). The groups were balanced for number (14), parity (3.0 ± 1.6 and 3.0 ± 1.8 for S + m and S groups, respectively), days in milk (151 ± 30 and 150 ± 26.4 days), and milk production (27.0 ± 4.7 and (27.3 ± 3.7 kg d^−1^ per head). Ingredients of the diets (kg per day) fed to lactating buffaloes were as follows: mycorrhizal silage (22 and 0 for S + m and S groups, respectively), nonmycorrhizal silage (0 and 22), meadow hay (6 and 6), concentrate (a mixture based on maize meal, soybean meal, sunflower meal, beet pulp, barley meal, wheat flour shorts, flaked maize, hydrogenated palm fat (14 and 14), and water (4 and 4)), and a mix of vitamins and minerals (0.4 and 0.4). The chemical composition of the diets expressed as g kg^−1^ of dry matter was as follows: crude protein 162 and 157, ether extract 40.3 and 40.7, neutral detergent fiber 420 and 425, acid detergent fiber 260 and 264, and UFL 850 and 850. Animal handling and milk sampling were like the maize test. Milk yield was 26.8 and 27.3 kg d^−1^ per head for the S + m and S groups, respectively.

### 4.4. Milk Sampling and Chemical Analysis

During the feeding tests, milk from each bovine was collected in sterile plain jars, kept at 4 °C, and sent to the laboratory for chemical analysis (MilkoScan FT 6000, Foss Electric, Hillerød, Denmark) to be conducted on the same day of collection. Milk collected in the morning and the evening was analyzed separately and average values were used for statistical analysis. Then, an aliquot of each daily sample was mixed and stored at −20 °C for fatty acid and volatile compound analysis. The use of inoculated silage did not affect the yield and chemical composition of milk in terms of fat, protein, and lactose content. The composition of milk from buffaloes fed maize silage, expressed as g kg^−1^, was as follows: fat 91 and 90, protein 45 and 45, and lactose 49 and 49 for the M + m and M groups, respectively. The composition of milk from cows fed sorghum silage, expressed as g kg^−1^, was as follows: fat 36 and 38, protein 34 and 33, and lactose 48 and 48 for the M + m and M groups, respectively.

### 4.5. Milk Fatty Acid Analysis

The extraction of fat from milk samples was carried out according to White et al. [50], with some modifications. The samples (5 g) were homogenized with ethanol (6.7 mL) and mixed using a Vortex mixer for 60 s. Then, a diethyl ether–heptane mixture (10 mL, 2:1 *v/v*) was added and mixed by vortexing for 60 s. Samples were then centrifuged at 3000 rpm for 10 min. The diethyl ether phase containing the extracted lipids was transferred and the residue was extracted 3 times using the same procedure. The filtrate was concentrated in a rotary evaporator at 36 °C. Then, the extracted lipid phase was dissolved in hexane and purified using sodium chloride saturated solution (3 mL). The hexane phase containing purified lipids was dried over anhydrous sodium sulfate and under nitrogen. Fatty acid methyl esters (FAMEs) were prepared using potassium hydroxide in a methanol solution (2N KOH in anhydrous MeOH), according to Ichihara et al. [51], with some modifications. Then 100 mg of extracted fat in a 10 mL glass-stoppered test-tube was dissolved in 2 mL n-hexane. A solution of 2 N potassium hydroxide in methanol (300 mL) was added. The tube was vortexed for 30 s and allowed to react for 4 min at room temperature. A 1 mL aliquot of the upper organic phase was analyzed by high-resolution gas chromatography. For the analysis, a Perkin Elmer Auto-system XL model gas chromatograph, equipped with a fused silica capillary column (100 m 0.25 mm i.d.; 0.20 µm film thickness; mod. SP 2380; Supelco Bellofonte, company, city, state abbreviation, USA), a flame ionization detector (FID), and a programmed temperature vaporizer (PTV), was used. The oven was set at an initial temperature of 100 °C for 5 min. The temperature was then increased at a rate of 3 °C min^−1^ to 165 °C and held for 10 min. This procedure was followed by a second increase in temperature at a rate of 3 °C min^−1^ to a final temperature of 260 °C, which was held for 28 min. The PTV operating conditions were 50 °C for 0.1 min, 400 °C min^−1^ to 260 °C, and held at 260 °C for 5 min. The split ratio selected was 1:30. The carrier gas was helium, with a linear velocity of 20 cm s^−1^. The FID conditions were a 10:1 ratio (air/hydrogen) and the temperature was 270 °C. The identification and quantification of separated peaks were performed using the Supelco 37 Component FAME MIX (Supelco Bellofonte, PA, USA), a CLA isomer mixture (Nu-Chek Prep., Inc. Elysian, MN, USA), as external standards, and GC retention data available in the literature. A CRM 164 milk fat reference supplied by the Community Bureau of Reference (Commission of the European Communities, Brussels, Belgium) was used to obtain response factors (Rfs) and convert areas of individual FAs into weight percentages of total fat. The FA analysis was performed in triplicate.

### 4.6. Milk Volatile Organic Compound Analysis

The extraction and analysis of VOCs was performed using dynamic headspace–solid phase micro-extraction (SPME) and GC/MS, according to Villeneuve et al. [18] and Lee et al. [52], with some modifications. First, 22.5 g of milk was transferred into a 50 mL bottle, then 30 µL of 2-methyl-3-heptanone, (99% purity, Sigma-Aldrich, St. Louis, MO, USA) as internal standard (408 mg L^−1^, in water solution), and 2.75 g of sodium phosphate (NaH_2_PO_4_) (Sigma-Aldrich), were added. The sample bottle was put in an apparatus that can regulate milk temperature and stirring of the sample. In order to homogenize it and accelerate the equilibrium of headspace-volatile compounds between the milk matrix and the headspace, the sample was magnetically stirred for 5 min at 55 °C. SPME fiber was inserted through the Teflon septum in the bottle and exposed to sample headspace for 60 min at 55 °C while stirring. The SPME device (Supelco Co., Bellefonte, state abbreviation, USA) was equipped with a 50/30 μm thick divinyl-benzene/carboxen/polydimethylsiloxane (DVB/CAR/PDMS) fiber coated with 2 cm length stationary phase. The same type of fiber was reported to be highly efficient for cheese aroma analysis [53]. Volatile compounds were analyzed by GC coupled with mass spectrometry using a Hewlett-Packard 6890 N (Agilent Technologies, Palo Alto, CA, USA), equipped with a J&W HP-5MS capillary column (30 m × 0.25 mm i.d. × 0.25 μm film thickness; J&W Scientific, Folsom, CA, USA). The temperature was set at 40 °C for 2 min and increased to 160 °C at the rate of 6 °C min^−1^ and to 210 °C at 10 °C min^−1^. The injector was kept at 250 °C. Helium was used as a carrier gas at 0.9 mL min^−1^. Volatile compound thermal desorption was carried out by exposing SPME fiber in the injector for 10 min. The identification of volatile compounds was carried out by comparing retention times and mass spectra obtained by analyzing pure reference compounds in the same conditions. The identification was confirmed by comparing mass spectra with those of the National Institute of Standards and Technology (NIST) database. All chemical standards were supplied by Sigma-Aldrich (St. Louis, MO, USA). In a few cases, the pure chemical standard was not available, and the compounds were labeled as tentative (t). Mass spectra were recorded at 70 eV. The source temperature was 230 °C and the interface temperature was 250 °C. Before use, the fiber was conditioned at 270 °C for 1 h for the analysis. In order to prevent the release of undesirable compounds, a blank test was performed before each analysis. The quantitative data of milk volatile compounds was carried out by normalizing the peak areas of each compound with respect to the area of the internal standard peak. Peak area data were processed by ChemStation software (Agilent Technologies). The analysis of volatile compounds was performed in triplicate.

### 4.7. Statistical Analysis of Data

Significant differences among the different samples were determined for each compound by one-way ANOVA statistical analysis with second-order interactions. Tukey’s test was used to discriminate among the means of the variables. The significance level was *p* ≤ 0.05 throughout the analyses. The data elaboration was carried out using XLStat (version 2009.3.02, Microsoft Corporation, Redmond, WA, USA), an add-in software package for Microsoft Excel (Addinsoft Corp., Paris, France).

## Figures and Tables

**Table 1:** Fatty acid composition of milk obtained from buffaloes fed nonmycorrhizal (M) and mycorrhizal (M + m) maize silage (% weight of total methyl esters; mean ± standard deviation). SFA, saturated fatty acid; MUFA, monounsaturated fatty acid; PUFA, polyunsaturated fatty acid.

Fatty Acids		M	sd	M + m	sd	Change (%) ^a^ M + m/M	
C4:0	Butyric acid	7.41	0.27	9.00	0.59	+22	*
C6:0	Caproic acid	3.64	0.07	4.77	0.45	+31	*
C8:0	Caprylic acid	1.91	0.26	2.21	0.19	+16	
C10:0	Capric acid	3.15	0.27	3.91	0.19	+24	*
C11:0	Undecanoic acid	0.07	0.01	0.07	0.01	+3	
C12:0	Lauric acid	3.74	0.26	4.37	0.325	+17	
C13:0	Tridecylic acid	0.16	0.01	0.15	0.01	−8	
C14:0	Myristic acid	14.11	0.39	14.82	0.035	+5	
C15:0	Pentadecylic acid	1.50	0.08	1.37	0.02	−9	
C16:0	Palmitic acid	39.49	1.23	37.94	0.09	−4	
C17:0	Margaric acid	0.49	0.01	0.39	0.03	−20	*
C18:0	Stearic acid	5.92	0.48	4.72	0.72	−20	
C24:0	Lignoceric acid	0.05	0.01	0.05	0.00	−16	
**SFA**		**81.64**	**0.15**	**83.8**	**1.02**	+3	*
C14:1n9	Myristoleic acid	1.60	0.13	1.70	0.00	+6	
C16:1n9	Palmitoleic acid	2.73	0.36	2.76	0.11	+1	
C17:1	Heptadecenoic acid	0.29	0.00	0.26	0.00	−12	*
C18:1n9t	Elaidic acid	0.25	0.01	0.38	0.19	+53	
C18:1n9c	Oleic acid	11.37	0.73	9.31	1.05	−18	*
C18:1n7	Vaccenic acid	0.16	0.01	0.12	0.02	−21	
C20:1n9	Eicosenoic acid	0.08	0.0	0.07	0.01	−15	
C22:1n9	Erucic acid	0.03	0.0	0.03	0.01	+6	
C24:1	Nervonic acid	0.06	0.01	0.05	0.0	−26	*
**MUFA**		**16.5**	**0.22**	**14.68**	**0.79**	−11	*
C18:2n6tc+ct	Trans-linoleic acid	0.21	0.02	0.14	0.01	−32	*
C18:2n6c	Linoleic acid	1.10	0.03	0.90	0.14	−19	
CLA	Conjugated linoleic acid	0.31	0.02	0.25	0.04	−17	
C20:3n6	Homo-γ-linoleic acid	0.04	0.00	0.04	0.01	−3	
C20:4n6	Arachidonic acid	0.10	0.01	0.10	0.02	+6	
C22:2n6	Docosadienoic acid	0.04	0.01	0.05	0.00	+4	
**PUFA n-6**		**1.59**	**0.06**	**1.34**	**0.24**	−16	
C18:3n3	α-Linolenic acid	0.13	0.00	0.10	0.01	−24	*
C20:5n3	Eicosapentaenoic acid (EPA)	0.02	0.00	0.02	0.00	+1	
C22:6n3	Docosahexaenoic acid (DHA)	0.04	0.01	0.03	0.00	−16	
**PUFA n-3**		**0.19**	**0.01**	**0.15**	**0.02**	−19	*
**ω6/ω3**		**8.42**	**0.15**	**8.74**	**0.67**	+4	

Asterisks indicate significantly different values between milk samples obtained from buffaloes fed on mycorrhizal and nonmycorrhizal maize silage (* *p* < 0.05). ^a^ Relative difference. The total saturated fatty acids (SFA), monounsaturated fatty acids (MUFA), polyunsaturated fatty acids (PUFAs) and the ratio **ω6/ω3** are shown in bold.

**Table 2:** Fatty acid composition (% of total fatty acids) of milk obtained from cows fed nonmycorrhizal (S) and mycorrhizal (S + m) sorghum silage (% weight of total methyl esters; mean ± standard deviation).

Fatty Acids		S	sd	S + m	sd	Change (%) ^a^ S+/S−	
C4:0	Butyric acid	5.53	0.86	7.69	0.96	+39	*
C6:0	Caproic acid	3.40	0.44	4.35	0.04	+28	*
C8:0	Caprylic acid	1.95	0.28	2.16	0.03	+11	
C10:0	Capric acid	4.25	0.64	4.17	0.48	−2	
C11:0	Undecanoic acid	0.11	0.04	0.09	0.02	−17	
C12:0	Lauric acid	4.79	0.71	4.64	0.39	−2	
C13:0	Tridecylic acid	0.17	0.04	0.17	0.01	−3	
C 14:0	Myristic acid	14.10	0.35	14.52	0.21	+3	
C15:0	Pentadecylic acid	1.43	0.11	1.43	0.00	0	
C16:0	Palmitic acid	37.94	0.76	38.78	0.99	+2	
C17:0	Margaric acid	0.50	0.02	0.43	0.06	−14	
C18:0	Stearic acid	5.82	0.86	4.86	0.52	−16	
C24:0	Lignoceric acid	0.06	0.00	0.05	0.01	−8	
**SFA**		**80.06**	**1.90**	**83.34**	**0.69**	+4	*
C14:1n9	Myristoleic acid	1.94	0.14	1.78	0.08	−8	
C16:1n9	Palmitoleic acid	1.88	0.17	2.34	0.58	+25	
C 17:1	Heptadecenoic acid	0.27	0.02	0.25	0.02	−9	
C18:1n9t	Elaidic acid	0.93	0.37	0.66	0.04	−29	
C18:1n9	Oleic acid	11.66	1.43	9.31	0.53	−20	
C18:1n7	Vaccenic acid	0.16	0.02	0.15	0.03	−6	
C22:1n9	Erucic acid	0.06	0.01	0.04	0.01	−24	
C24:1	Nervonic acid	0.06	0.01	0.04	0.01	−51	*
**MUFA**		**16.95**	**1.47**	**14.56**	**0.14**	−14	*
C18:2n6tc+ct	Trans-linoleic acid	0.27	0.07	0.19	0.07	−29	
C18:2n6c	Linoleic acid	1.73	0.20	1.17	0.29	−32	
CLA	Conjugated linoleic acid	0.40	0.09	0.28	0.04	−31	
C20:3n6	Homo-γ-linoleic acid	0.10	0.03	0.28	0.04	+21	
C22:2n6	Docosadienoic acid	0.05	0.00	0.05	0.00	−8	
**PUFA n-6**		**2.55**	**0.35**	**1.81**	**0.40**	−29	
C18:3n3	α-Linolenic acid	0.35	0.07	0.23	0.13	−32	
C20:5n3	EPA	0.02	0.00	0.01	0.00	−35	
C22:6n3	DHA	0.07	0.01	0.04	0.01	−44	*
**PUFA n-3**		**0.44**	**0.08**	**0.29**	**0.14**	−34	
**ω6/ω3**		**5.88**	**0.26**	**7.49**	**3.31**	+27	

Asterisks indicate significantly different values between milk samples obtained from cows fed on mycorrhizal and nonmycorrhizal sorghum silage (* *p* < 0.05). ^a^ Relative difference. The total saturated fatty acids (SFA), monounsaturated fatty acids (MUFA), polyunsaturated fatty acids (PUFAs) and the ratio **ω6/ω3** are shown in bold.

**Table 3:** Headspace concentrations (μg kg^−1^) and odor descriptors of volatile compounds determined in milk samples obtained from buffaloes fed nonmycorrhizal (M) and mycorrhizal (M + m) maize silage.

Compound	Odor Descriptors ^a^	Concentration		Change (%) M + m vs. M ^b^	
		M	M + m		
Ketones					
Acetone		55.82 ± 12.22	26.14 ± 2.67	−53	*
2-Butanone	Varnish [35]	16.08 ± 4.05	18.85 ± 1.89	+17	
2-Heptanone	Animals [35], blue cheese [35,36,37,38,39], moldy [36], spicy [37,38,39], cinnamon [37,38], musty [40], varnish [40], sweet [40], Roquefort cheese [39]	8.04 ± 1.57	18.85 ± 1.58	+134	*
2-Nonanone	Hot milk [35], smoked cheese [35], varnish [36], fruity [37,38,40], floral [37,38,40], peachy [41]	4.75 ± 0.48	7.07 ± 0.22	+46	*
Acids					
Butanoic acid	Cheese [40,41,42,43,44], rotten [40], sharp [40], rancid cheese [39], putrid [39], vomit [16,41], sweaty [39,44], buttery [42], fermented [42], fecal [43,44], cheese [16]	22.75 ± 2.63	28.96 ± 1.65	+27	*
Hexanoic acid	Sharp [40], goaty [40,44], pungent [39], blue cheese [39], sour [39]	165.18 ± 3.14	172.77 ± 41.34	+5	
Octanoic acid	Goaty [39,42], waxy [39,42], soapy [39], musty [39], rancid [39,42], fruity [39], unpleasant [42], fatty [42], body odor [43,44], sweat 43,44]	87.23 ± 14.80	130.06 ± 9.79	+49	*
Decanoic acid	Waxy-sweet [40], rancid fatty [39]	18.50 ± 2.53	23.75 ± 1.67	+28	*
Undecanoic acid ^(t)^		6.65 ± 1.47	6.39 ± 0.43	−4	
Tetradecanoic acid ^(t)^		8.23 ± 0.96	8.15 ± 0.96	−1	
Esters					
Ethyl acetate	Sticky [45], sweet [45]	7.60 ± 0.52	nf	–	*
Alcohols					
2-Ethyl hexanol ^(t)^	Spicy [35]	9.80 ± 1.05	10.49 ± 0.60	+7	
Aldehydes					
Hexanal	Green apple [45], grassy [45]	6.61 ± 0.60	7.02 ± 1.20	+6	
Nonanal	Green [35,36,37,46], animals [35,36,46], grass-like [36,37,46], fatty [36,37,46], floral [37], citrus [37,41]	10.65 ± 1.51	20.03 ± 1.19	+88	*
Decanal		nf	3.67 ± 0.26	+	*

nf, not found. ^(t)^ Volatile compounds were tentatively identified. Asterisks indicate significant differences between mycorrhizal and nonmycorrhizal samples (* *p* < 0.05). ^a^ Sensory descriptors reported for volatile compounds are from literature data. ^b^ Relative difference.

**Table 4:** Headspace concentrations (μg kg^−1^) and odor descriptors of volatile compounds determined in milk samples obtained from cows fed nonmycorrhizal (S) and mycorrhizal (S + m) sorghum silage.

Compound	Odor Descriptors ^a^	Concentration		Change (%) S + m vs. S ^b^	
		S	S+m		
Ketones					
Acetone		5.38 ± 1.32	5.49 ± 1.27	+2	
2-Butanone	Varnish [35]	5.06 ± 0.08	4.19 ± 0.14	−17	*
2-Nonanone	Hot milk [35], smoked cheese [35], varnish [36], fruity [37,38,39], floral [37,38,40], peachy [40]	1.65 ± 0.09	3.11 ± 0.58	+88	*
Acids					
Butanoic acid	Cheese [40,41,42,43,44], rotten [40], sharp [40], rancid cheese [39], putrid [39], vomit [16,41], sweaty [39,44], buttery [42], fermented [42], fecal [43,44], cheese [16]	7.23 ± 1.40	27.43 ± 2.60	+279	*
Hexanoic acid	Sharp [40], goaty [40,44], pungent [39], blue cheese [39], sour [39]	88.70 ± 14.16	180.81 ± 32.45	+104	*
Octanoic acid	Goaty [39,42], waxy [39,42], soapy [39], musty [39], rancid [39,42], fruity [39], unpleasant [42], fatty [42], body odor [43,44], sweat [43,44]	91.66 ± 5.40	190.11 ± 42.92	+107	*
Nonanoic acid		nf	2.17 ± 0.36	+	
Decanoic acid	Waxy-sweet [40], rancid fatty [39]	31.47 ± 1.41	60.71 ± 2.45	+93	*
Undecanoic acid ^(t)^		3.57 ± 0.51	6.34 ± 0.07	+78	*
Tetradecanoic acid ^(t)^		2.20 ± 0.72	3.01 ± 0.21	+37	*
Esters					
Ethyl acetate	Sticky [45], sweet [45]	2.89 ± 0.02	nf	-	*
Alcohols					
2-Ethyl hexanol ^(t)^	Spicy [35]	15.36 ± 0.67	14.99 ± 1.07	−2	
Aldehydes					
Hexanal	Green apple [45], grassy [45]	16.22 ± 2.39	11.92 ± 2.35	−27	
Nonanal	Green [35,36,37,46], animals [35,36,46], grass-like [36,37,46], fatty [36,37,46], floral [37], citrus [37,41]	7.91 ± 1.77	7.36 ± 0.34	−7	
Decanal		2.31 ± 0.44	2.96 ± 0.65	−28	

nf, not found. ^(t)^ Volatile compounds were tentatively identified. Asterisks indicate significant differences between mycorrhizal and nonmycorrhizal samples (* *p* < 0.05). ^a^ Sensory descriptors reported for volatile compounds are from literature data. ^b^ Relative difference.
